# The hidden link: Investigating functional connectivity of rarely explored sub-regions of thalamus and superior temporal gyrus in Schizophrenia

**DOI:** 10.1515/tnsci-2022-0356

**Published:** 2024-12-11

**Authors:** Adnan Alahmadi, Jamaan Al-Ghamdi, Haythum O. Tayeb

**Affiliations:** Radiologic Sciences Department, Faculty of Applied Medical Sciences, King Abdulaziz University, Jeddah, 21589, Saudi Arabia; Radiologic Sciences Department, Faculty of Applied Medical Sciences, King Abdulaziz University, Jeddah, Saudi Arabia; Faculty of Medicine, King Abdulaziz University, Jeddah, Saudi Arabia; Faculty of Medicine in Rabigh, King Abdulaziz University, Jeddah, Saudi Arabia

**Keywords:** schizophrenia, fMRI, functional connectivity, superior temporal gyrus, sub-thalamic regions

## Abstract

Functional magnetic resonance imaging (fMRI) stands as a pivotal tool in advancing our comprehension of Schizophrenia, offering insights into functional segregations and integrations. Previous investigations employing either task-based or resting-state fMRI primarily focused on large main regions of interest (ROI), revealing the thalamus and superior temporal gyrus (STG) as prominently affected areas. Recent studies, however, unveiled the cytoarchitectural intricacies within these regions, prompting a more nuanced exploration. In this study, resting-state fMRI was conducted on 72 schizophrenic patients and 74 healthy controls to discern whether distinct thalamic nuclei and STG sub-regions exhibit varied functional integrational connectivity to main networks and to identify the most affected sub-regions in Schizophrenia. Employing seed-based analysis, six sub-ROIs – four in the thalamus and two in the STG – were selected. Our findings unveiled heightened positive functional connectivity in Schizophrenic patients, particularly toward the anterior STG (aSTG) and posterior STG (pSTG). Notably, positive connectivity emerged between the medial division of mediodorsal thalamic nuclei (MDm) and the visual network, while increased functional connectivity linked the ventral lateral nucleus of the thalamus with aSTG. This accentuated functional connectivity potentially influences these sub-regions, contributing to dysfunctions and manifesting symptoms such as language and learning difficulties alongside hallucinations. This study underscores the importance of delineating sub-regional dynamics to enhance our understanding of the nuanced neural alterations in Schizophrenia, paving the way for more targeted interventions and therapeutic approaches.

## 1 Introduction

Schizophrenia is a complex neuropsychiatric disorder characterized by a constellation of symptoms, including delusions, hallucinations, impaired emotional reactivity, social withdrawal, and cognitive dysfunction. These symptoms significantly impair psychosocial functioning and pose a considerable burden on individuals, their families, and society at large. While the pathogenesis of schizophrenia remains incompletely understood, emerging evidence suggests it is a neurodevelopmental disorder influenced by genetic, developmental, and environmental factors. These factors contribute to structural and functional alterations in the brain’s neural circuitry, particularly affecting thalamic connectivity, which is crucial to the disorder’s symptomatology [1,2].

Dysfunction in thalamic connectivity is increasingly recognized as central to the neural circuit abnormalities observed in schizophrenia [3]. The thalamus, a crucial subcortical structure, plays a vital role in sensory processing, motor functions, and cognitive and emotional processing. It comprises over 60 interconnected nuclei that form extensive circuits with both cortical and subcortical regions [4,5]. Notably, alterations in thalamic volume and connectivity, particularly with the prefrontal cortex, somatosensory regions, cerebellum, and temporal cortex, have been documented in schizophrenia [6,7,8,9,10,11,12,13,14,15,16,17,18]. However, these studies often consider the thalamus as a whole, overlooking the distinct roles and connectivity patterns of its individual nuclei [19]. Similarly, the superior temporal gyrus (STG) is implicated in schizophrenia, especially concerning auditory hallucinations and cognitive deficits [20,21,22,23,24,25,26,27,28]. Yet, research has not adequately differentiated the functions and connectivity of the STG’s cytoarchitectonic subregions.

Our study aims to address these gaps by focusing on the functional connectivity of specific, underexplored thalamic nuclei and STG subregions in individuals with schizophrenia. This approach is grounded in the premise that the unique anatomical and functional characteristics of these subregions may underlie specific aspects of the disorder’s symptomatology. Our selection of the mediodorsal thalamic nuclei (MDm), ventral anterior (VA) nucleus, anterior pulvinar nucleus (PuA), ventral lateral (VL) nucleus of the thalamus, and the anterior and posterior subregions of the STG (aSTG and pSTG) is based on their potential relevance to the diverse symptoms of schizophrenia, as highlighted in prior research and our preliminary findings. For instance, the MDm’s role in the thalamo-prefrontal cortical circuitry is crucial for cognitive control and emotional processing, which are core aspects disrupted in schizophrenia. The MDm’s connectivity with the prefrontal cortex underpins its involvement in executive function and working memory, facets profoundly affected in schizophrenia, as underscored by recent studies [29,30]. The VA and VL nuclei, integral to motor control and connected with the premotor and motor cortices, are relevant to schizophrenia beyond mere anatomical associations, given the disorder’s link with motor dysfunctions. Our examination of VA and VL aims to shed light on their potential contributions to schizophrenia’s motor symptoms, informed by research emphasizing the thalamic regions’ significance in motor functions [31]. The PuA, linked to somatosensory regions, plays a role in sensory integration, which is often compromised in schizophrenia. Including PuA in our study allows us to investigate its involvement in the disorder’s altered sensory processing, as discussed in research on thalamic contributions to sensory and cognitive functions [32]. The aSTG and pSTG are crucial for language and social cognition, domains where deficits are commonly seen in schizophrenia. Exploring these STG subregions can offer insights into the neural underpinnings of schizophrenia-related communication and social interaction challenges, with support from studies highlighting the cortical regions’ importance in cognitive and social functions [33].

The rationale for focusing on these specific thalamic nuclei and STG subregions, despite the broader involvement of various brain regions in schizophrenia, is derived from their documented deviations in schizophrenia patients and their lack of representation in existing studies [34]. Our decision to explore the designated thalamic and STG subfields is based on their pivotal roles in cognitive control, motor function, emotional regulation, and sensory processing – all commonly disrupted in schizophrenia. Thus, our study’s objectives are refined to investigate the resting-state functional connectivity of these selected thalamic nuclei and STG subregions with the entire brain, aiming to elucidate their differential roles and connectivity patterns in schizophrenia. In doing so, we aspire to contribute novel insights into the neural foundations of schizophrenia and identify potential therapeutic targets, leveraging the comprehensive dataset from The Center for Biomedical Research Excellence (COBRE).

## 2 Materials and methods

### 2.1 Subject recruitment and scanning

The dataset of this study consists of 72 patients with schizophrenia (60 male, right-handed, age ranged 18–65 years) and 74 healthy controls (51 male, right-handed, age ranged 18–65 years). Patients with a history of neurological disorder, history of mental retardation, history of severe head trauma with more than 5 min loss of consciousness, and a history of substance abuse within the last 12 months were excluded. The data were downloaded from The COBRE, which is a non-restricted public dataset and is available freely. This dataset is a part of the International Neuroimaging Data-Sharing Initiative under the 1000 Functional Connectomes Project. Full Information on this dataset is available at http://cobre.mrn.org/as well as in the following studies [35–38]. COBRE is a publically available dataset distributed with “Creative Commons License: Attribution – Non-Commercial,” and written informed consent was obtained from all subjects in accordance with the institutional review board (IRB) protocols of the University.

All the participants were scanned with a Siemens TIM 3.0 Tesla scanner. A multi-echo MPRAGE (MEMPR) sequence was used with the following parameters: TR/TE/TI = 2,530/[1.64, 3.5, 5.36, 7.22, 9.08]/900 ms, flip angle = 7°, FOV = 256 mm × 256 mm, slab thickness = 176 mm, matrix = 256 × 256 × 176, voxel size = 1 mm × 1 mm × 1 mm, number of echoes = 5, pixel bandwidth = 650 Hz, and total scan time = 6 min. Using five echoes, the TR, TI, and time to encode partitions for the MEMPR are similar to that of a conventional MPRAGE, resulting in similar GM/WM/CSF contrast. Resting-state functional magnetic resonance imaging (rs-fMRI) data were collected with single-shot full k-space echo-planar imaging (EPI) with ramp sampling correction using the intercomissural line (AC-PC) as a reference (TR: 2 s, TE: 29 ms, matrix size: 64 × 64, 32 slices, and voxel size: 3 × 3 × 4 mm^3^). During the acquisition process, all subjects were instructed to keep their eyes open and stare at the fixation cross.

### 2.2 rs-fMRI preprocessing

The functional data preprocessing was conducted using the SPM12 and CONN toolbox implemented on MATLAB [39]. Initially, the first few volumes of each functional MRI dataset were discarded to allow for signal stabilization and reduce the impact of initial transient effects. Skull stripping was performed on both functional and structural images to remove non-brain tissues. Motion correction was applied to account for head movements during scanning, and the functional images were realigned and unwrapped.

The T1-weighted structural images were segmented into gray matter, white matter, and cerebrospinal fluid (CSF) regions, providing valuable information for further analyses. The functional images were then normalized to the standard Montreal Neurological Institute (MNI) space using the structural data. Temporal band-pass filtering was applied to retain signals within the desired frequency range while minimizing noise.

Additionally, the global signal was regressed out to further reduce confounding effects. Outlier correction was performed using the Artifact Detection Tools implemented in CONN to detect and correct data outliers. Finally, the images were smoothed using an 8-mm full-width half-maximum (FWHM) isotropic Gaussian kernel. Temporal processing with data denoising was also carried out to neutralize the effects of artifacts and confounding parameters from the BOLD signal in regions of no interest.

### 2.3 ROI selections

This study was conducted with the aim of investigating the connectivity between selected seed regions and broader brain functional networks, utilizing rs-fMRI as the method of analysis. A total of six seeds, situated bilaterally across the hemispheres, were chosen for this analysis. These seeds comprise the medial division of the medidorsal thalamic nuclei (MDm), the VA nucleus of the thalamus, the PuA of the thalamus, the VL nucleus of the thalamus, the aSTG, and the pSTG. To outline the scope of our investigation, Table 1 details the targeted networks and ROI. These selections were rigorously defined, employing a combination of tools such as cytoarchitectonic probability anatomy maps and the Automated Anatomical Labelling Atlas 3 [40–44], ensuring a precise and scientifically grounded selection process. The rationale behind the seed selection was their clear identification within the atlas.

**Table 1:** Demographics of all subjects

Group	Gender	Count	Mean age (years)	Std age (years)	Min age (years)	Max age (years)	Median age (years)	Right-handed count	Left-handed count	Both-handed count
Healthy	Male	51	36.4	11.8	18	65	35	49	1	1
	Female	23	34.5	11.1	18	58	33	22	0	1
Patients	Male	58	37.5	14	18	64	36	48	8	2
	Female	14	40.9	13.5	20	65	40.5	12	2	0

In addition to the seeds, our study also focused on a series of targeted brain regions, encompassing a wide array of functional networks. These were meticulously identified and are documented in Table 2, based on criteria set forth in the CONN toolbox. Among these networks are the default mode, attention, sensorimotor, visual, salience, dorsal attention, frontoparietal, cerebellar, and language networks. The delineation of these networks was obtained using an independent component analysis (ICA) conducted on data from 497 participants in the Human Connectome Project.

**Table 2:** Targeted selected networks with the regions that constitute them; the coordinates of these regions are also shown

Network	Region (sub-region)	MNI coordinates (*x*, *y*, *z*)	Key findings
Default mode	Medial prefrontal cortex (MPFC)	(1, 55, −3)	Increased connectivity with MDm and VA nuclei in schizophrenia patients; disrupted connectivity patterns with sensorimotor networks
	Lateral parietal (LP) left	(−39, −77, 33)	Higher connectivity with visual networks in schizophrenia patients
	LP right	(47, −67, 29),	Similar pattern as left LP with stronger connectivity in schizophrenia
	Posterior cingulate cortex (PCC)	(1, −61, 38)	Altered connectivity patterns with both MDm and VA nuclei in schizophrenia patients
Sensorimotor	Lateral left	(−55, −12, 29)	Elevated connectivity with anterior and posterior thalamic nuclei in schizophrenia patients
	Lateral right	(56, −10, 29)	Similar to left lateral sensorimotor with stronger connectivity in schizophrenia patients
	Superior	(0, −31, 67)	Increased connectivity with lateral sensorimotor and visual networks in schizophrenia patients
Visual	Medial	(2, −79, 12)	Altered connectivity in schizophrenia patients compared to controls
	Occipital	(0, −93, −4)	No significant differences in connectivity between schizophrenia patients and healthy controls
	Lateral left	(−37, −79, 10)	Altered connectivity patterns with other visual regions in schizophrenia patients
	Lateral right	(38, −72, 13)	Similar pattern as left lateral visual with connectivity alterations
Salience	Anterior cingulate cortex (ACC)	(0, 22, 35)	Reduced connectivity with MDm and VA nuclei in schizophrenia patients
	Insula left	(−44, 13, 1)	Disrupted connectivity with default mode and visual networks in schizophrenia patients
	Insula right	(47, 14, 0)	Similar pattern to left insula with altered connectivity in schizophrenia patients
	Rostral lateral prefrontal cortex (RPFC) left	(−32, 45, 27)	Reduced connectivity with thalamic nuclei, particularly in VL and MDm, in schizophrenia patients
	RPFC right	(32, 46, 27)	Similar pattern as left RPFC with connectivity deficits in schizophrenia patients
	Supramarginal gyrus (SMG) left	(−60, −39, 31)	Connectivity disruptions affecting both sensorimotor and visual networks in schizophrenia patients
	SMG right	(62, −35, 32)	Similar disruptions as observed in the left hemisphere
Dorsal attention	Frontal eye fields (FEF) left	(−27, −9, 64)	Reduced connectivity with visual and sensorimotor networks in schizophrenia patients
	FEF right	(30, −6, 64)	Similar to left FEF, showing reduced connectivity in schizophrenia patients
	Intraparietal sulcus (IPS) left	(−39, −43, 52)	Altered connectivity affecting visual-spatial processing in schizophrenia patients
	IPS right	(39, −42, 54)	Similar pattern as left IPS with connectivity changes in schizophrenia patients
Fronto-Parietal	Lateral prefrontal cortex (LPFC) left	(−43, 33, 28)	Disrupted connectivity with thalamic nuclei, affecting cognitive functions in schizophrenia patients
	Posterior parietal cortex (PPC) left	(−46, −58, 49)	Elevated connectivity with visual networks in schizophrenia patients
	LPFC right	(41, 38, 30)	Similar pattern as left LPFC with connectivity alterations in schizophrenia patients
	PPC right	(52, −52, 45)	Similar disruptions observed in the left hemisphere
Language	Inferior frontal gyrus (IFG) left	(−51, 26, 2)	Increased connectivity with thalamic and cerebellar networks in schizophrenia patients
	IFG right	(54, 28, 1)	Similar pattern to left IFG with stronger connectivity in schizophrenia patients
	pSTG left	(−57, −47, 15)	Elevated connectivity with thalamic nuclei, especially MDm and VL, in schizophrenia patients
	pSTG right	(59, −42, 13)	Similar pattern to left pSTG with no significant differences in healthy controls
Cerebellar	Anterior	(0, −63, −30)	Increased connectivity with thalamic and sensorimotor networks in schizophrenia patients
	Posterior	(0, −79, −32)	Similar pattern as anterior cerebellar network with no significant changes in healthy controls

### 2.4 Statistical analysis

Statistical analyses were performed on two distinct levels. Initially, at the individual subject level, the analysis involved the use of weighted bivariate correlation models applying general linear methods, focusing on connectivity matrices that describe interactions between predefined ROIs. These matrices were generated based on the Fisher-transformed bivariate correlation coefficients for ROI pairs’ time-series data. Subsequently, at a broader scale, the analysis was used to assess and contrast the functional connectivity metrics at a collective level. This step aimed to discern and differentiate the rs-fMRI networks associated with various sub-regions across subjects. Results were standardized employing a correction for the false discovery rate (FDR) set at *p* < 0.05, adhering to multivariate statistics through a parametric analysis approach (MVPA omnibus test) [39,45]. In this context, inferences at the cluster level were made by leveraging multivariate parametric statistical inferences on functional network connectivity, taking into account clusters or networks comprising interconnected ROIs. The process entailed a comprehensive evaluation of all inter-ROI connections, examining both internal and external network connectivity [46]. By applying a multivariate parametric general linear model to all connections, this approach facilitated a detailed analysis. The output is a map depicting F-statistical tests for each network pair. The FDR threshold for clusters signifies the ratio of expected false discoveries among network pairs showing equivalent or more significant effects within the entire array of functional connectivity network pairs [47]. Preferring FDR over the family-wise error rate is advantageous for its enhanced sensitivity in peak detection while minimizing the likelihood of false positives [48]. For further information, refer to the mentioned articles or visit the CONN toolbox website: https://web.conn-toolbox.org.


**Ethical approval:** The research related to human use has complied with all the relevant national regulations and institutional policies in accordance with the tenets of the Helsinki Declaration and has been approved by the authors’ IRB or equivalent committee. This study was approved by the Faculty of Applied Medical Sciences Committee (code 01-2022 – Date of Approval 07-01-2022).
**Informed consent:** Informed consent has been obtained from all individuals included in this study.

## 3 Results

**Figure 1:**
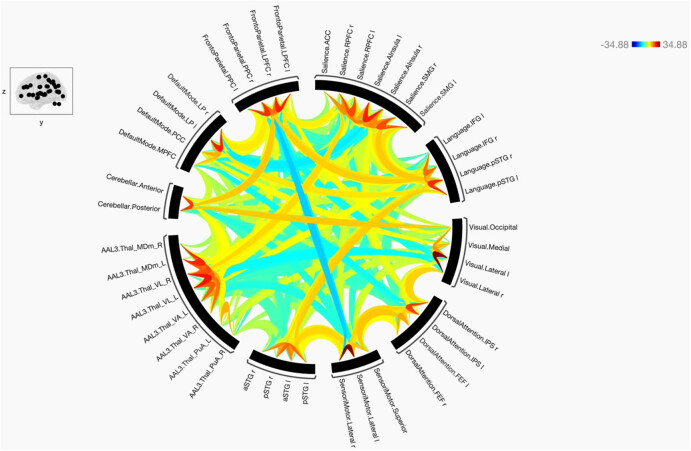
The functional connectivity between chosen seeds and target areas demonstrated at the collective level in healthy individuals. Red lines signify positive connections, whereas blue lines represent negative ones. The intensity of the line colors reflects the statistical significance, with the T-bar displayed in the upper right corner.

**Figure 2:**
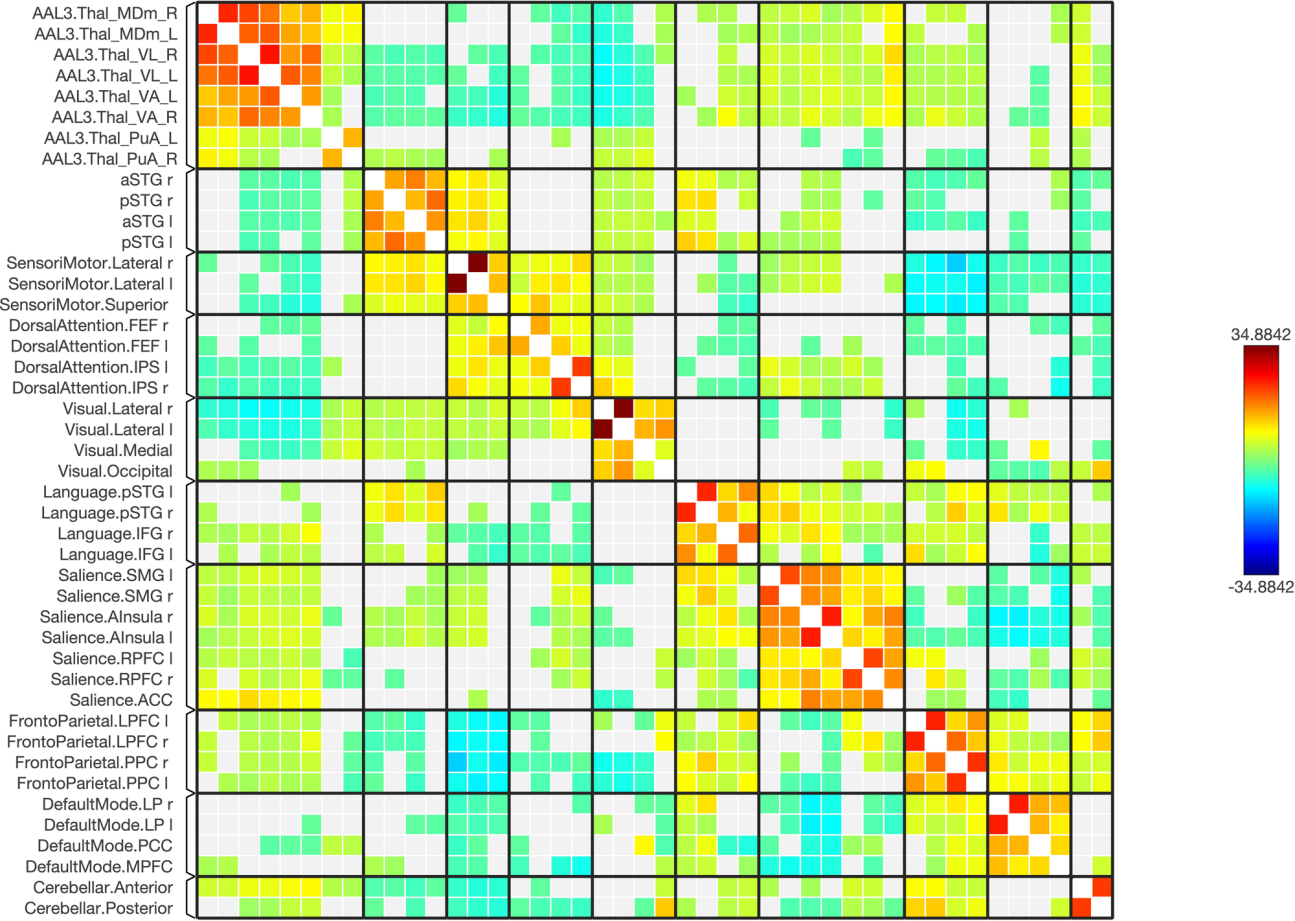
A connectivity matrix in healthy subjects, featuring the chosen seeds (the first 12 rows) and the targeted neural networks, including statistical metrics of connection strength on the left. Right: Presents another matrix, where the connectivity levels are adjusted by a statistical normalization threshold to clarify distinctions between strong, weak, and negative connections for illustrative purposes.

**Figure 3:**
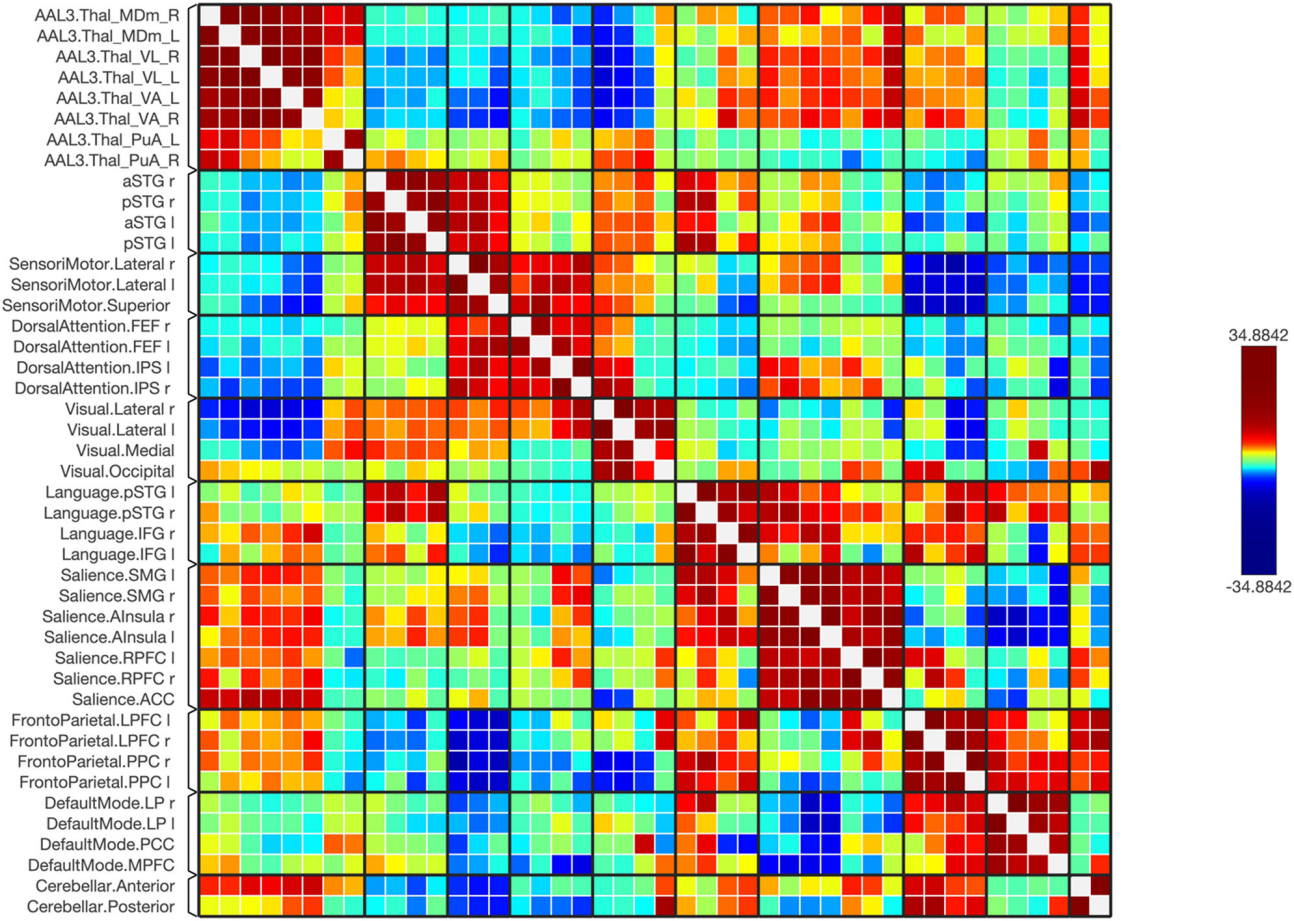
A matrix of the healthy subjects, where the connectivity levels are adjusted by a statistical normalization threshold to clarify distinctions between strong, weak, and negative connections for illustrative purposes.

**Figure 4:**
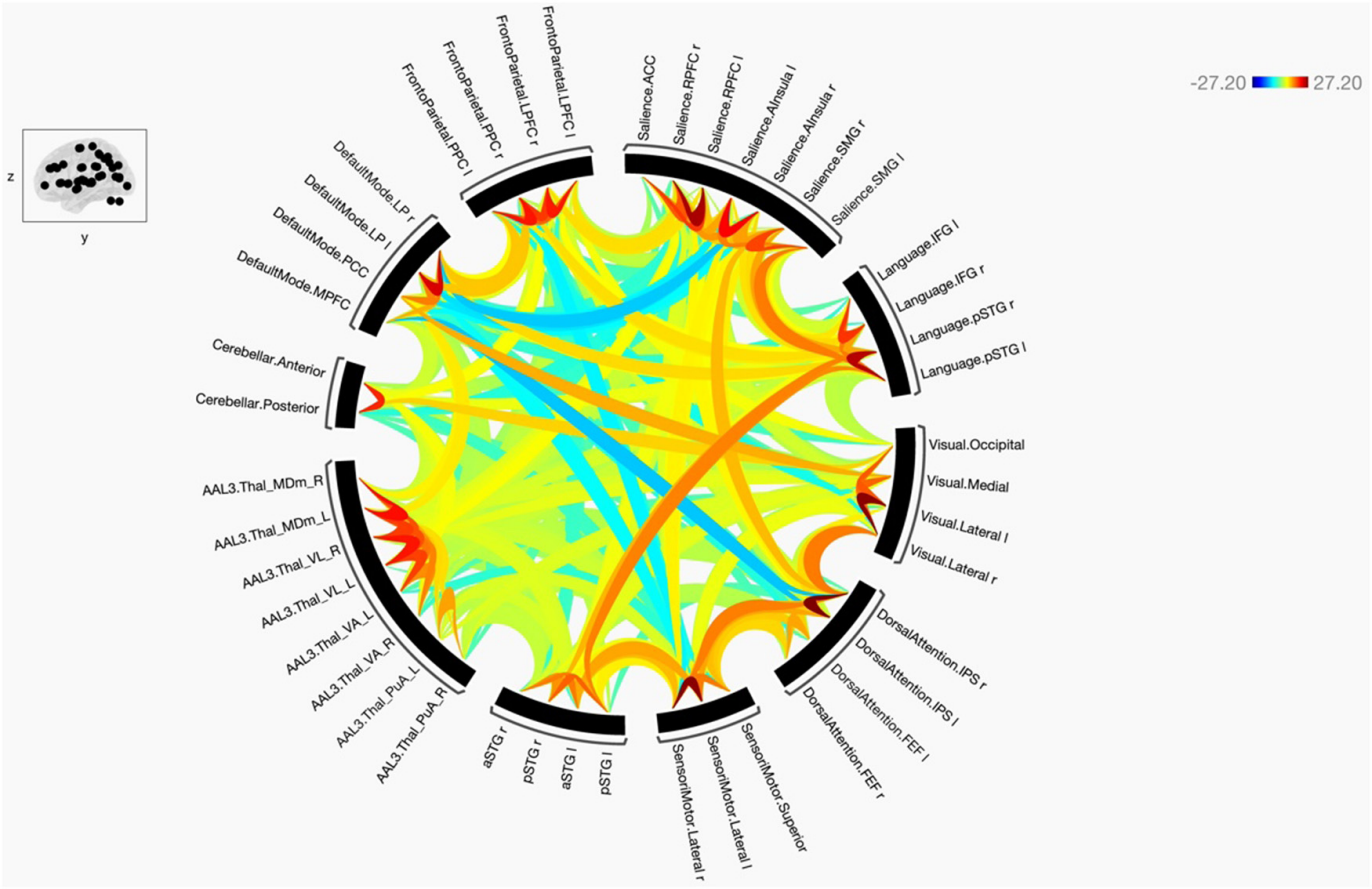
The functional connectivity between selected seeds and target regions at a group level in subjects with schizophrenia. Positive connections are marked with red lines, and negative connections are marked with blue lines. The line colors vary according to the statistical significance, and the T-bar is located in the top right-hand corner.

**Figure 5:**
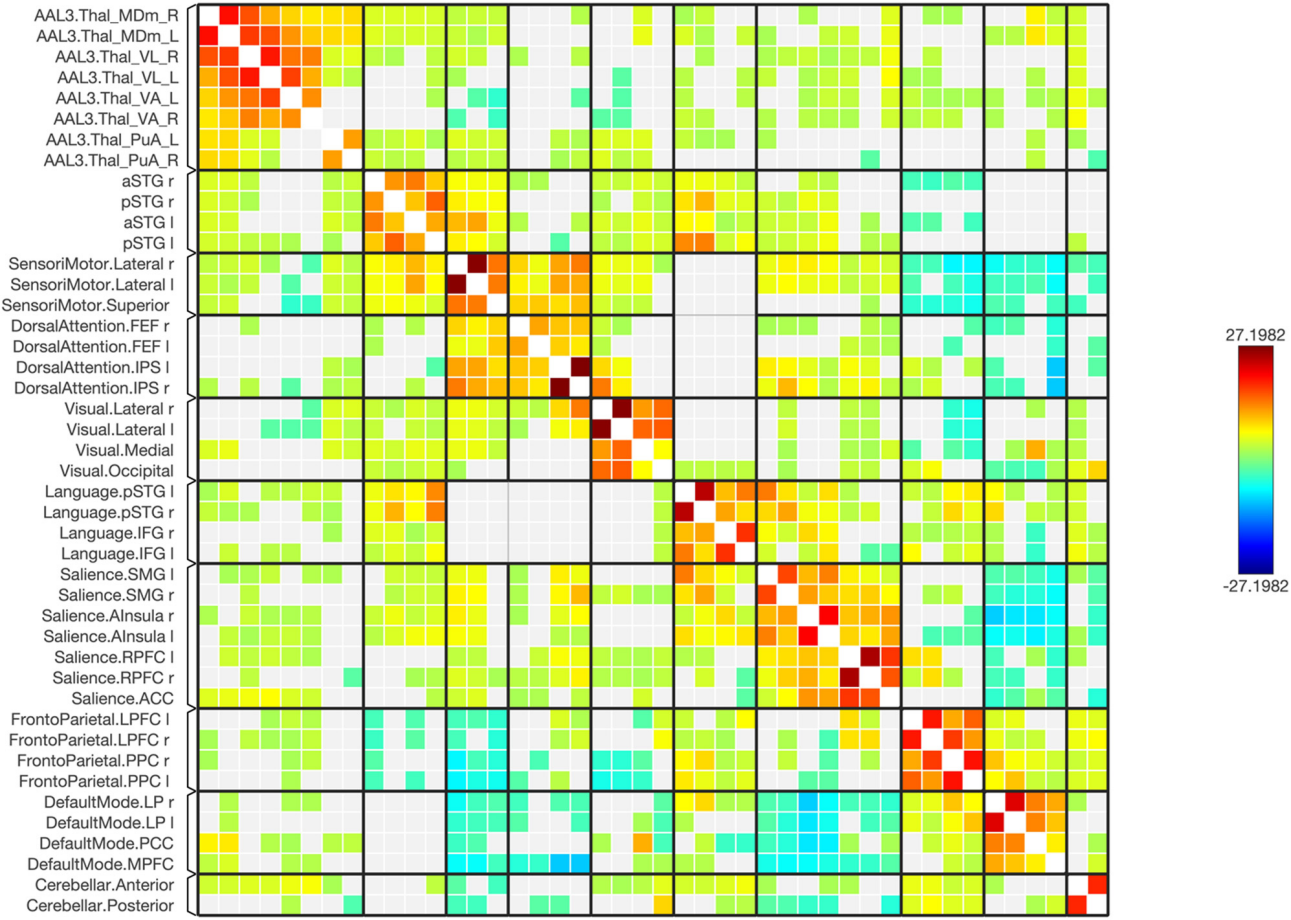
The matrix of the functional connectivity in the schizophrenic subjects of the selected seeds (top 12 rows) and the targeted brain networks is shown along with the statistical indications of the strength of the connection (left).

**Figure 6:**
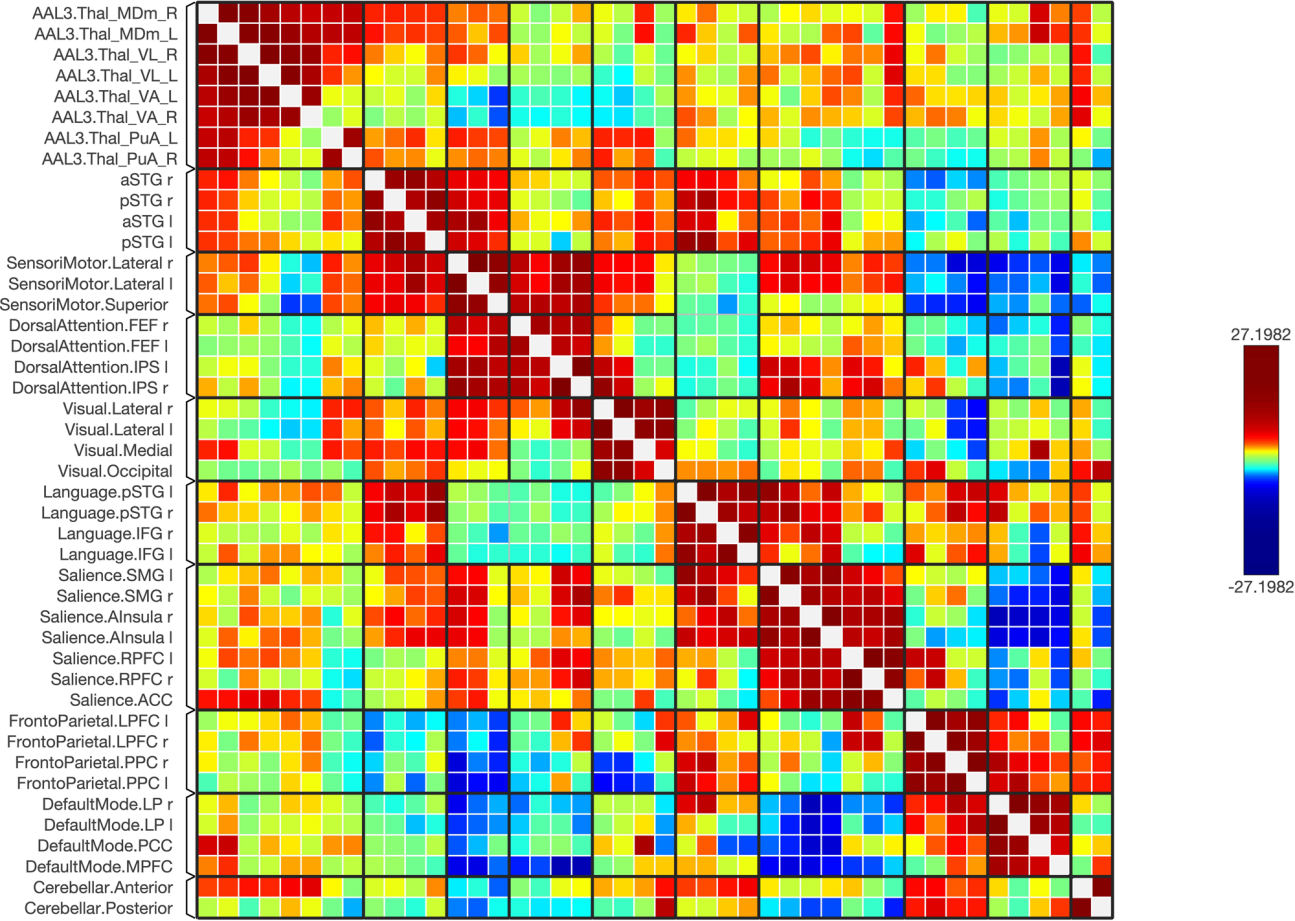
A matrix of the schizophrenic subjects; the matrix of the connectivity was set with a statistical equalizer threshold to easily distinguish strong, weak, and negative connections for illustration.

**Figure 7:**
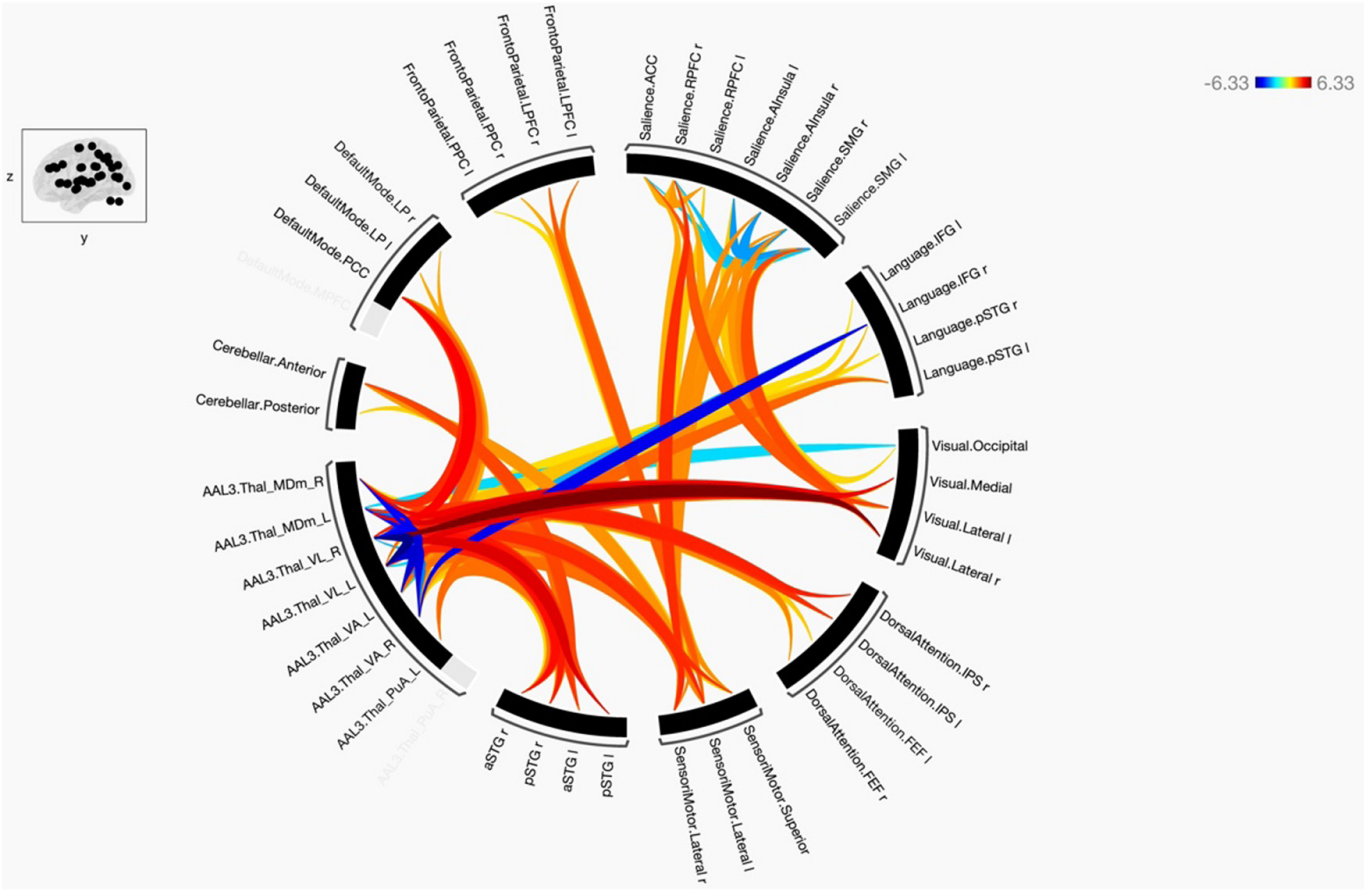
The functional connectivity of the selected seeds and target regions is shown at the group level in the direct comparison between schizophrenic > control subjects. The lines of the connections in red indicate +ve means SCz > HC, and blue −ve means HC > SCz. The colors of the lines are proportional to statistical strength, and the T-bar is shown in the top right-hand corner.

**Figure 8:**
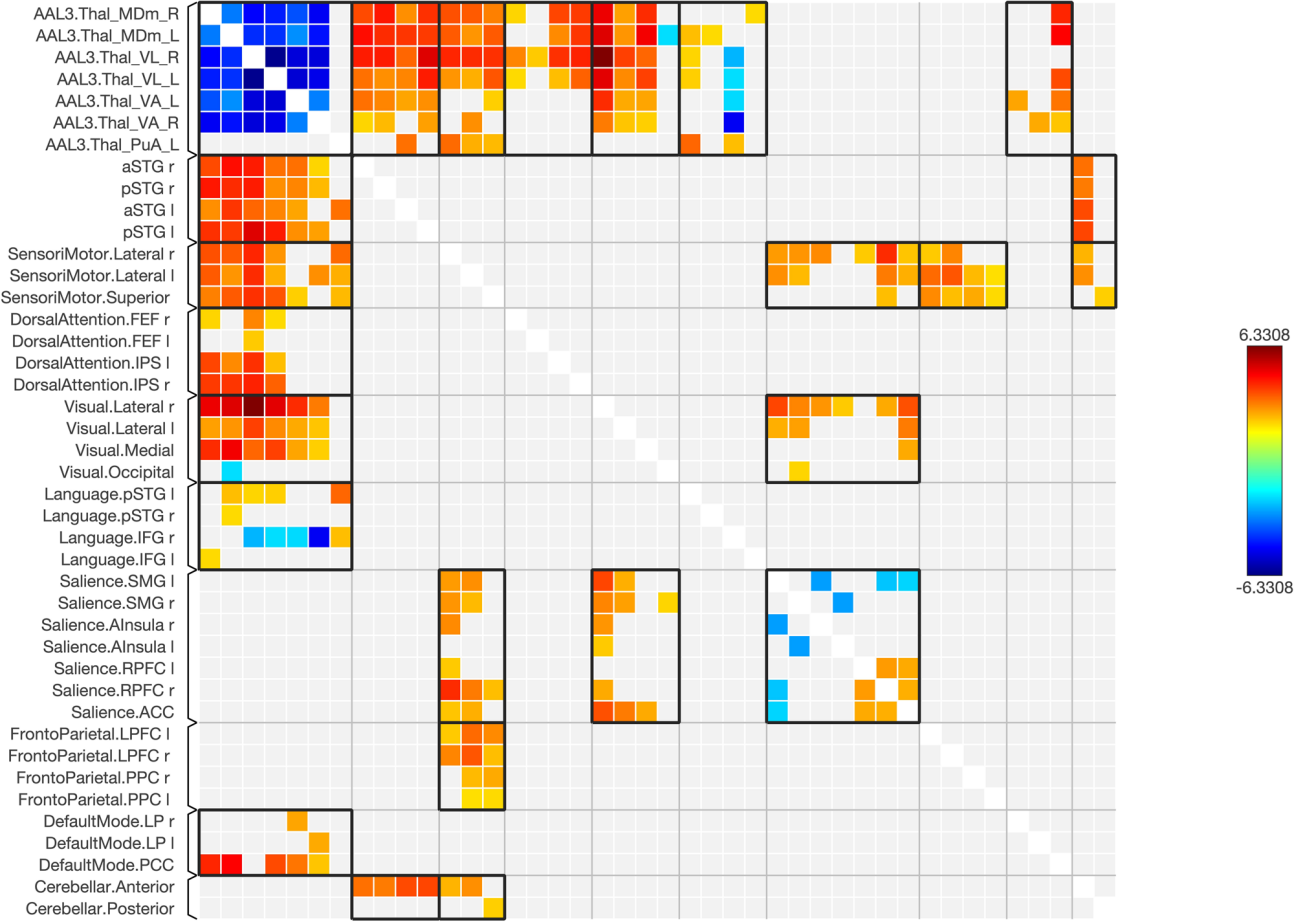
The matrix of the functional connectivity in the schizophrenic > control subjects of the selected seeds (top 12 rows) and the targeted brain networks is shown along with the statistical indications of the strength of the connection (left). Right: the matrix of the connectivity was set with a statistical equalizer threshold to easily distinguish strong, weak, and negative connections for illustration.

**Figure 9:**
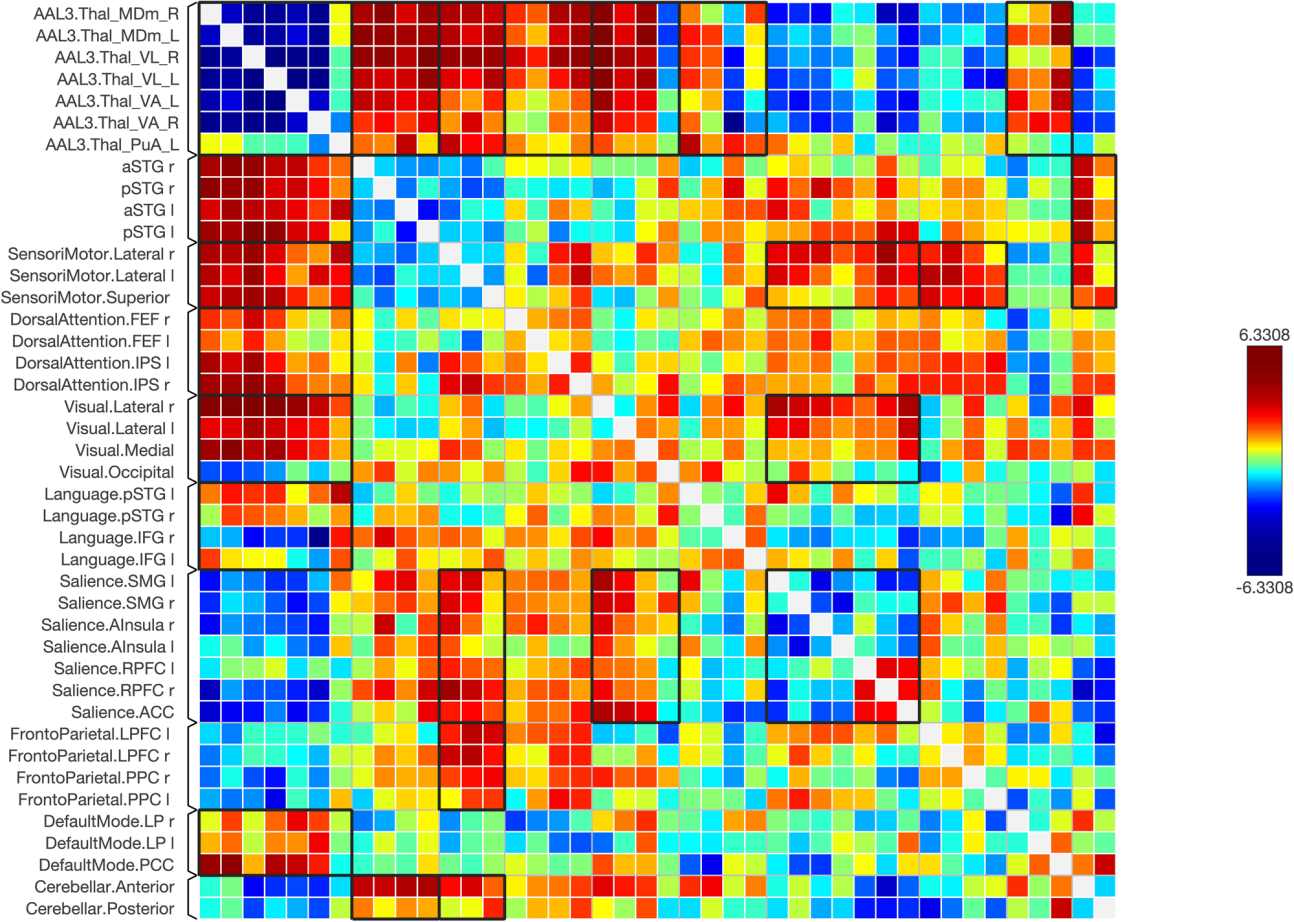
The matrix of the functional connectivity in the schizophrenic > control subjects of the selected seeds (top 12 rows) and the targeted brain networks is shown. Also, the matrix of the connectivity was set with a statistical equalizer threshold to easily distinguish strong, weak, and negative connections for illustration.

Functional connectivity between and among the ROIs of the healthy and schizophrenic subjects is shown in Figures 1–6, respectively. The figures show the results as a ring of functional connectivity and matrix connectivity. A summary of direct comparisons between connectivity in patients with schizophrenia and healthy controls is shown in Figures 7–9. A summary of the connected networks for each ROI is given next. A summary of key findings is also shown in Table 3.

**Table 3:** Targeted selected networks with the regions that constitute them and key findings

Network	Regions	Key findings
Default mode	MPFC	Increased connectivity with MDm and VA nuclei in schizophrenia patients; disrupted connectivity patterns with sensorimotor networks
	LP (L)	Higher connectivity with visual networks in schizophrenia patients
	LP (R)	Similar pattern as the left LP with stronger connectivity in schizophrenia
	PCC	Altered connectivity patterns with both MDm and VA nuclei in schizophrenia patients
Sensorimotor	Medial	Increased connectivity with lateral sensorimotor and visual networks in schizophrenia patients
	Occipital	No significant differences in connectivity between schizophrenia patients and healthy controls
	Lateral (L)	Elevated connectivity with anterior and posterior thalamic nuclei in schizophrenia patients
	Lateral (R)	Similar to the left lateral sensorimotor with stronger connectivity in schizophrenia patients
Salience	ACC	Reduced connectivity with MDm and VA nuclei in schizophrenia patients
	Insula (L)	Disrupted connectivity with default mode and visual networks in schizophrenia patients
	Insula (R)	Similar pattern to the left insula with altered connectivity in schizophrenia patients
	RPFC (L)	Reduced connectivity with thalamic nuclei, particularly in the VL and MDm, in schizophrenia patients
	RPFC (R)	Similar pattern as the left RPFC with connectivity deficits in schizophrenia patients
	SMG (L)	Connectivity disruptions affecting both sensorimotor and visual networks in schizophrenia patients
	SMG (R)	Similar disruptions as observed in the left hemisphere
Dorsal Attention	FEF (L)	Reduced connectivity with visual and sensorimotor networks in schizophrenia patients
	FEF (R)	Similar to the left FEF, showing reduced connectivity in schizophrenia patients
	IPS (L)	Altered connectivity affecting visual-spatial processing in schizophrenia patients
	IPS (R)	Similar pattern as the left IPS with connectivity changes in schizophrenia patients
Fronto-parietal	LPFC (L)	Disrupted connectivity with thalamic nuclei, affecting cognitive functions in schizophrenia patients
	PPC (L)	Elevated connectivity with visual networks in schizophrenia patients
	LPFC (R)	Similar pattern as the left LPFC with connectivity alterations in schizophrenia patients
	PPC (R)	Similar disruptions observed in the left hemisphere
Language	IFG (L)	Increased connectivity with thalamic and cerebellar networks in schizophrenia patients
	IFG (R)	Similar pattern to the left IFG with stronger connectivity in schizophrenia patients
	pSTG (L)	Elevated connectivity with thalamic nuclei, especially MDm and VL, in schizophrenia patients
	pSTG (R)	Similar pattern to the left pSTG with no significant differences in healthy controls
Cerebellar	Anterior	Increased connectivity with thalamic and sensorimotor networks in schizophrenia patients
	Posterior	Similar pattern as the anterior cerebellar network with no significant changes in healthy controls

### 3.1 Connectivity of the medial division of mediodorsal thalamic nuclei (MDm)

Using the right MDm as a seed, functional connectivity to the following regions was higher in schizophrenia patients compared to healthy controls: bilateral aSTG, bilateral pSTG, the right lateral visual networks (LVN), the right medial visual network (MVN), the right dorsal attention network, bilateral sensorimotor and default mode networks (DMN). In contrast, the following regions had lower connectivity with the right MDm in schizophrenia patients than healthy controls: the left MDm, bilateral VL thalamic nuclei, and bilateral VA nuclei. When looking at the left MDm as a seed, functional connectivity was higher to the following regions in schizophrenia patients compared to healthy subjects: bilateral aSTG, bilateral pSTG, the left LVN, the left MVN, and DMN. In contrast, connectivity was lower in schizophrenia patients in comparison to healthy controls in the following regions: the right MDm, bilateral VL nuclei, bilateral VA nuclei, and the visual occipital network.

### 3.2 Connectivity of the VA thalamic nucleus

When looking at the right VA nucleus as a seed, functional connectivity was higher in schizophrenia patients compared to healthy subjects in the following regions: the left pSTG, bilateral LVNs, and DMNs. On the other hand, connectivity was lower in schizophrenia patients compared to healthy controls in the following regions: bilateral MDm, bilateral VL nucleus, and the left VA nucleus. Using the left VA as a seed, functional connectivity was higher in schizophrenia patients compared to healthy subjects in the following regions: bilateral pSTG, bilateral aSTG, and DMNs. Connectivity was, in contrast, lower in schizophrenia in comparison to healthy subjects to bilateral MDm, bilateral VL nuclei, and the right VA nucleus.

### 3.3 Connectivity of the PuA

When looking at the left PuA as a seed, functional connectivity was mostly higher in schizophrenia patients compared to healthy subjects. This included the bilateral lateral sensorimotor networks. There is no significantly higher connectivity in healthy subjects compared to schizophrenia patients. The right PuA demonstrates no significant higher connectivity in healthy subjects compared to schizophrenia patients and schizophrenia patients compared to healthy subjects.

### 3.4 Connectivity of the VL nucleus

When looking at the right VL as a seed, functional connectivity to the following regions was higher in schizophrenia patients than healthy subjects: bilateral pSTG, bilateral aSTG, sensorimotor superior and lateral networks, the right MVN, and right LVN. Lower connectivity was seen in the following regions in schizophrenia patients compared to healthy subjects: the right VL nucleus, bilateral VA nuclei, and bilateral MDm nuclei. Using the left VL as a seed, functional connectivity was higher in the following regions in schizophrenia patients compared to healthy subjects: bilateral pSTG, bilateral aSTG, sensorimotor superior and lateral networks, the MVN, and the LVN. Lower connectivity in schizophrenia patients in comparison to healthy subjects was found in the following regions: the right VL nucleus, bilateral VA nuclei, and bilateral MDm nuclei.

### 3.5 Connectivity of the aSTG

When looking at the right aSTG as a seed, functional connectivity to the following regions was higher in schizophrenia patients compared to healthy subjects: bilateral MDm nuclei, bilateral VL nuclei, the left VA nucleus, and anterior cerebellar networks. Using the left aSTG as a seed, functional connectivity to the following regions was lower in schizophrenia patients compared to healthy subjects: bilateral MDm nuclei, bilateral VL nuclei, the left VA nucleus, and anterior cerebellar networks.

### 3.6 Connectivity of the pSTG

When looking at the right pSTG as a seed, functional connectivity to the following regions was higher in schizophrenia patients compared to healthy subjects: bilateral MDm nuclei, bilateral VL nuclei, bilateral VA nuclei, and the anterior cerebellar network. There were no regions with significantly lower connectivity to the right pSTG in schizophrenia patients in comparison to healthy subjects. Using the left pSTG as a seed, functional connectivity to the following regions was higher in schizophrenia patients compared to healthy subjects: bilateral MDm nuclei, bilateral VL nuclei, bilateral VA nuclei, and the anterior cerebellar network. There were no regions with significantly lower connectivity with the pSTG in schizophrenia patients in comparison to healthy subjects.

## 4 Discussion

This investigation into schizophrenia’s neural underpinnings through fMRI data highlights diminished functional connectivity within select thalamic sub-regions (MDm, VA, PuA, and VL) compared to healthy controls. This reduction in connectivity, previously underexplored, suggests disruptions in thalamocortical loops and informational integration, potentially impacting thalamocortical and cortico-striato-thalamo-cortical circuits’ role in differentiating internal from external perceptions, a key mechanism in reality construction and symptom manifestation in schizophrenia [49,50,51,52,53,54,55,56,57,58,59,60,61,62,63,64,65,66].

Our findings align with theories proposing that altered thalamic connectivity affects brain oscillatory activities, impacting the brain’s ability to modulate these activities based on sensory input. The presence of low-frequency oscillations during wakefulness, typically restricted to slow-wave sleep, supports this, suggesting a link between these neural activities and the under-constrained perception characteristic of schizophrenia’s hallucinatory experiences [67,68,69,70,71,72,73].

Consistent with prior research, our study observed increased connectivity between the thalamus and networks such as the DMN, sensorimotor, visual, and cerebellar networks. This enhanced thalamic engagement with sensorimotor regions, previously associated with schizophrenia’s positive symptoms, underscores the potential role of neural integration disruptions in symptomatology [10,12,19,74,75,76,77,78,79,80]. The DMN’s heightened connectivity, implicated in self-referential thoughts and daydreaming, could relate to schizophrenia’s reality distortion symptoms [81,82,83].

Particularly noteworthy is the increased connectivity between the VL and STG, suggesting potential auditory processing disruptions linked to cognitive difficulties in schizophrenia. This aligns with temporal lobe abnormalities associated with the disorder’s positive symptoms [84].

The study further explores the role of specific thalamic nuclei in schizophrenia’s diverse symptoms, emphasizing the importance of thalamic projections in behaviors ranging from arousal to cognitive functions [85]. The mediodorsal thalamic nuclei (MDm) connections with limbic and prefrontal areas and its role in cognitive control highlight the complex interplay between thalamic dysfunction and schizophrenia’s cognitive and emotional symptoms [19,30,31,77,86,87,88,89,90,91,92,93,94,95,96,97].

Similarly, the STG’s involvement in auditory processing and language, coupled with its hyperconnectivity with the MDm, underscores the potential neural basis of auditory hallucinations and related symptoms in schizophrenia [22,23,65,66]. The STG’s role in social cognition further implicates dysconnectivity in the broader spectrum of schizophrenia’s symptoms, including thought disorder and social dysfunction [98,99].

Our study’s interpretations of rs-fMRI FC patterns, suggesting direct associations between thalamic connectivity and specific brain functions or symptoms in schizophrenia, must be approached with caution. The complexity of brain connectivity, involving potentially multisynaptic pathways rather than direct monosynaptic connections, challenges straightforward correlations between observed FC alterations and clinical manifestations of schizophrenia. For instance, the inferred impact of thalamic sub-regions like the VL nucleus on auditory processing areas such as the STG does not account for the lack of direct anatomical connections, underscoring the speculative nature of such conclusions.

Furthermore, the suggestion that decreased intra-thalamic FC reflects disruptions in thalamocortical loops and information integration may overestimate our findings’ specificity. Given the absence of monosynaptic connections between thalamic nuclei, the observed FC likely represents complex, multisynaptic networks, complicating the direct attribution of these connectivity patterns to specific dysfunctions in schizophrenia.

This study’s exploration of schizophrenia’s neural mechanisms through rs-fMRI highlights critical limitations due to the spatial resolution and data smoothing techniques employed. With a resolution of 3 mm × 3 mm × 4 mm, capturing the detailed connectivity of thalamic nuclei, known for their small size, proves challenging. Spatial smoothing, intended to enhance signal-to-noise ratio (SNR) and adjust for anatomical variations, might further obscure the delicate signals from these crucial areas. Although aimed at improving data analysis, such smoothing risks diluting the specificity of connectivity signals, particularly for small brain structures. Future research should pivot toward utilizing higher-resolution rs-fMRI data and refining imaging techniques to improve spatial specificity without sacrificing SNR or extending scan times. Adjusting the approach to spatial smoothing or avoiding it when analyzing small brain regions could yield a more accurate depiction of functional connectivity patterns. Enhancing ROI definition methods will also be vital for improving study outcomes.

This study, investigating the neural mechanisms of schizophrenia through rs-fMRI, acknowledges the methodological limitations imposed by the spatial resolution and data smoothing techniques utilized. Employing a resolution of 3 mm × 3 mm × 4 mm and 8 mm FWHM smoothing aimed to enhance the SNR for analyzing large-scale brain networks but presented challenges in capturing the intricate connectivity of smaller brain structures like thalamic nuclei. The AAL3 atlas demands high-resolution data to fully leverage its detailed parcellation, especially in areas like the thalamus, where precision is crucial for accurate analysis.

While the smoothing and resolution settings were beneficial for broader network analyses, they may not provide the necessary spatial precision for detailed identification and analysis of smaller structures, potentially obscuring the nuanced signals essential for understanding schizophrenia’s neural basis. Future research should consider higher-resolution rs-fMRI data and refined imaging techniques to improve spatial specificity without compromising SNR or extending scan times. Adjusting spatial smoothing strategies or avoiding them when analyzing small brain regions could enhance the accuracy of functional connectivity patterns. Enhancing ROI definition methods will also be critical for advancing study outcomes, allowing for a more nuanced exploration of the brain’s connectivity and function. Despite these methodological challenges, it is important to note that the findings of this study still contribute valuable insights into the neural underpinnings of schizophrenia, adding to the broader understanding of this complex condition. The study’s results, while limited by the aforementioned constraints, provide important findings that enrich our knowledge and set the groundwork for future research to build upon, ensuring that neuroimaging research continues to evolve in its capacity to dissect the intricate details of brain function and connectivity.

Moreover, the study’s reliance on an open-access dataset introduces significant limitations in the availability of clinical and demographic details, particularly concerning the selection and characterization of the schizophrenic cohort. The lack of detailed participant information, such as the length of time since diagnosis, specific treatments received, and the presence of other health conditions, restricts our ability to comprehensively link connectivity patterns with schizophrenia symptoms or patient histories. Additionally, the absence of precise definitions for the ‘healthy’ cohort further complicates the interpretation of our findings. These limitations highlight the importance of collecting detailed clinical data, including factors like age, gender, medication history, and other health conditions, which are known to influence brain connectivity. Addressing these methodological and data-related challenges in future studies, including refining the definitions of “schizophrenic” and “healthy” cohorts, will be crucial for advancing our understanding of schizophrenia. Emphasizing the collection of detailed clinical data and employing advanced neuroimaging techniques could foster more nuanced insights into the disorder, potentially informing therapeutic strategies.

While our study focused on the functional connectivity differences between schizophrenia patients and healthy controls, we recognize the potential influence of gender and age differences, which were part of the recruitment regimen. However, our current analysis did not reveal significant findings related to these variables. The broad age range and the inclusion of both male and female participants could introduce variability in connectivity patterns that we were unable to fully explore within the scope of this study. Future research should aim to investigate these factors more thoroughly, as they may offer valuable insights into the heterogeneity of schizophrenia and contribute to a more personalized understanding of the disorder. Including a more detailed analysis of gender-specific and age-related connectivity differences could enhance our understanding of how these demographic variables interact with the neurobiological mechanisms underlying schizophrenia.

While our current study included participants with varying handedness, our variability test suggested no significant impact of handedness on the functional connectivity outcomes, likely due to the small number of left-handed and ambidextrous individuals in our sample. However, handedness is known to influence brain connectivity patterns, and its potential effects should not be overlooked. Future research should consider conducting analyses on larger, more balanced groups of right-handed, left-handed, and ambidextrous participants to better understand how handedness may affect neurobiological mechanisms in schizophrenia. Additionally, excluding non-right-handed participants or separately analyzing them could provide clearer insights and strengthen the validity of the results.

## 5 Conclusion

In summary, this research delves into the less explored sub-regions of the thalamus, including the medial division of the mediodorsal thalamic nuclei (MDm), the VA nucleus, the VL nucleus, and the left PuA. We discovered that patients with schizophrenia exhibit increased connectivity within these thalamic subdivisions. Furthermore, both the aSTG and pSTG showed enhanced connectivity with these thalamic sub-regions, marking the highest deviation observed in our study. Such abnormal connectivity patterns, particularly in the thalamus and STG, may underpin the hallucinations and cognitive difficulties frequently encountered in schizophrenia.

## References

[1] Cannon TD, van Erp TG, Bearden CE, Loewy R, Thompson P, Toga AW, et al. Early and late neurodevelopmental influences in the prodrome to schizophrenia: contributions of genes, environment, and their interactions. Schizophr Bull. 2003;29(4):653–69. 10.1093/oxfordjournals.schbul.a007037 14989405

[2] Lewis DA, Levitt P. Schizophrenia as a disorder of neurodevelopment. Annu Rev Neurosci. 2002;25:409–32. 10.1146/annurev.neuro.25.112701.142754 12052915

[3] Sigurdsson T. Neural circuit dysfunction in schizophrenia: insights from animal models. Neuroscience. 2016;321:42–65. 10.1016/j.neuroscience.2015.06.059 26151679

[4] Wang HL, Rau CL, Li YM, Chen YP, Yu R. Disrupted thalamic resting-state functional networks in schizophrenia. Front Behav Neurosci. 2015;9:45. 10.3389/fnbeh.2015.00045 PMC4340165 25762911

[5] Fama R, Sullivan EV. Thalamic structures and associated cognitive functions: Relations with age and aging. Neurosci Biobehav Rev. 2015;54:29–37. 10.1016/j.neubiorev.2015.03.008 PMC4457546 25862940

[6] Gröhn C, Norgren E, Eriksson L. A systematic review of the neural correlates of multisensory integration in schizophrenia. Schizophr Res: Cognit. 2022;27:100219. 10.1016/j.scog.2021.100219 PMC8502765 34660211

[7] van Erp TG, Hibar DP, Rasmussen JM, Glahn DC, Pearlson GD, Andreassen OA, et al. Subcortical brain volume abnormalities in 2028 individuals with schizophrenia and 2540 healthy controls via the ENIGMA consortium. Mol Psychiatry. 2016;21(4):547–53. 10.1038/mp.2015.63 PMC4668237 26033243

[8] Faria AV, Zhao Y, Ye C, Hsu J, Yang K, Cifuentes E, et al. Multimodal MRI assessment for first episode psychosis: A major change in the thalamus and an efficient stratification of a subgroup. Hum Brain Mapp. 2021;42(4):1034–53. 10.1002/hbm.25276 PMC7856640 33377594

[9] Okada N, Fukunaga M, Yamashita F, Koshiyama D, Yamamori H, Ohi K, et al. Abnormal asymmetries in subcortical brain volume in schizophrenia. Mol Psychiatry. 2016;21(10):1460–6. 10.1038/mp.2015.209 PMC5030462 26782053

[10] Zhang M, Palaniyappan L, Deng M, Zhang W, Pan Y, Fan Z, et al. Abnormal thalamocortical circuit in adolescents with early-onset schizophrenia. J Am Acad Child Adolesc Psychiatry. 2021;60(4):479–89. 10.1016/j.jaac.2020.07.903 32791099

[11] Ramsay IS. An activation likelihood estimate meta-analysis of thalamocortical dysconnectivity in psychosis. Biol Psychiatry: Cognit Neurosci Neuroimaging. 2019;4(10):859–69. 10.1016/j.bpsc.2019.04.007 31202821

[12] Giraldo-Chica M, Woodward ND. Review of thalamocortical resting-state fMRI studies in schizophrenia. Schizophr Res. 2017;180:58–63. 10.1016/j.schres.2016.08.005 PMC5297399 27531067

[13] Culbreth AJ, Wu Q, Chen S, Adhikari BM, Hong LE, Gold JM, et al. Temporal-thalamic and cingulo-opercular connectivity in people with schizophrenia. NeuroImage: Clin. 2021;29:102531. 10.1016/j.nicl.2020.102531 PMC7750447 33340977

[14] Ferri J, Ford JM, Roach BJ, Turner JA, van Erp TG, Voyvodic J, et al. Resting-state thalamic dysconnectivity in schizophrenia and relationships with symptoms. Psychol Med. 2018;48(15):2492–9. 10.1017/S003329171800003X PMC12094034 29444726

[15] Fryer SL, Ferri JM, Roach BJ, Loewy RL, Stuart BK, Anticevic A, et al. Thalamic dysconnectivity in the psychosis risk syndrome and early illness schizophrenia. Psychol Med. 2022;52(13):2767–75. 10.1017/S0033291720004882 33719985

[16] Dorph-Petersen K-A, Lewis DA. Postmortem structural studies of the thalamus in schizophrenia. Schizophr Res. 2017;180:28–35. 10.1016/j.schres.2016.08.007 PMC5746188 27567291

[17] Gong J, Luo C, Li X, Jiang S, Khundrakpam BS, Duan M, et al. Evaluation of functional connectivity in subdivisions of the thalamus in schizophrenia. Br J Psychiatry. 2019;214(5):288–96. 10.1192/bjp.2018.299 30791964

[18] Diedrichsen J, Balsters JH, Flavell J, Cussans E, Ramnani N. A probabilistic MR atlas of the human cerebellum. NeuroImage. 2009;46(1):39–46. 10.1016/j.neuroimage.2009.01.045 19457380

[19] Anticevic A, Cole MW, Repovs G, Murray JD, Brumbaugh MS, Winkler AM, et al. Characterizing thalamo-cortical disturbances in schizophrenia and bipolar illness. Cereb Cortex. 2014;24(12):3116–30. 10.1093/cercor/bht165 PMC4224238 23825317

[20] Steullet P. Thalamus-related anomalies as candidate mechanism-based biomarkers for psychosis. Schizophr Res. 2020;226:147–57. 10.1016/j.schres.2019.05.027 31147286

[21] Hazlett EA, Buchsbaum MS, Byne W, Wei TC, Spiegel-Cohen J, Geneve C, et al. Three-dimensional analysis with MRI and PET of the size, shape, and function of the thalamus in the schizophrenia spectrum. Am J Psychiatry. 1999;156(8):1190–9. 10.1176/ajp.156.8.1190 10450259

[22] Kasai K, Shenton ME, Salisbury DF, Hirayasu Y, Lee CU, Ciszewski AA, et al. Progressive decrease of left superior temporal gyrus gray matter volume in patients with first-episode schizophrenia. Am J Psychiatry. 2003;160(1):156–64. 10.1176/appi.ajp.160.1.156 PMC2845847 12505815

[23] Narayanaswamy JC, Kalmady SV, Venkatasubramanian G, Gangadhar BN. Clinical correlates of superior temporal gyrus volume abnormalities in antipsychotic-naive schizophrenia. J Neuropsychiatry Clin Neurosci. 2015;27(2):e128–33. 10.1176/appi.neuropsych.14030049 25923856

[24] Zhou B, Tan C, Tang J, Chen X. Brain functional connectivity of functional magnetic resonance imaging of patients with early-onset schizophrenia. Zhong Nan Da Xue Xue Bao Yi Xue Ban. 2010;35(1):17–24. 10.3969/j.issn.1672-7347.2010.01.003 20130360

[25] Ohi K, Matsuda Y, Shimada T, Yasuyama T, Oshima K, Sawai K, et al. Structural alterations of the superior temporal gyrus in schizophrenia: detailed subregional differences. Eur Psychiatry. 2016;35:25–31. 10.1016/j.eurpsy.2016.02.002 27061374

[26] Hoptman MJ, Zuo XN, Butler PD, Javitt DC, D'Angelo D, Mauro CJ, et al. Amplitude of low-frequency oscillations in schizophrenia: a resting state fMRI study. Schizophr Res. 2010;117(1):13–20. 10.1016/j.schres.2009.09.030 PMC2822110 19854028

[27] Orlov ND, Giampietro V, O'Daly O, Lam SL, Barker GJ, Rubia K, et al. Real-time fMRI neurofeedback to down-regulate superior temporal gyrus activity in patients with schizophrenia and auditory hallucinations: a proof-of-concept study. Transl Psychiatry. 2018;8(1):1–10. 10.1038/s41398-017-0067-5 PMC5865171 29430009

[28] Pearlson GD. Superior temporal gyrus and planum temporale in schizophrenia: a selective review. Prog Neuro-psychopharmacol Biol Psychiatry. 1997;21(8):1203–29. 10.1016/s0278-5846(97)00159-0 9460087

[29] Parnaudeau S, Bolkan SS, Kellendonk C. The mediodorsal thalamus: An essential partner of the prefrontal cortex for cognition. Biol Psychiatry. 2018;83(8):648–56. 10.1016/j.biopsych.2017.11.008 PMC5862748 29275841

[30] DeNicola AL, Park MY, Crowe DA, MacDonald AW, Chafee MV. Differential roles of mediodorsal nucleus of the thalamus and prefrontal cortex in decision-making and state representation in a cognitive control task measuring deficits in Schizophrenia. J Neurosci. 2020;40(8):1650–67. 10.1523/JNEUROSCI.1703-19.2020 PMC7046322 31941665

[31] Pergola G, Danet L, Pitel AL, Carlesimo GA, Segobin S, Pariente J, et al. The regulatory role of the human mediodorsal thalamus. Trends Cognit Sci. 2018;22(11):1011–25. 10.1016/j.tics.2018.08.006 PMC6198112 30236489

[32] Copeland CS, Neale SA, Salt TE. Neuronal activity patterns in the mediodorsal thalamus and related cognitive circuits are modulated by metabotropic glutamate receptors. Neuropharmacology. 2015;92:16–24. 10.1016/j.neuropharm.2014.12.031 PMC4362770 25576798

[33] Ouhaz Z, Fleming H, Mitchell AS. Cognitive functions and neurodevelopmental disorders involving the prefrontal cortex and mediodorsal thalamus. Front Neurosci. 2018;12:33. 10.3389/fnins.2018.00033 PMC5808198 29467603

[34] Kaur A, Basavanagowda DM, Rathod B, Mishra N, Fuad S, Nosher S, et al. Structural and functional alterations of the temporal lobe in schizophrenia: A literature review. Cureus. 2020;12(10):e11177. 10.7759/cureus.11177 PMC7689947 33262914

[35] Mayer AR, Ruhl D, Merideth F, Ling J, Hanlon FM, Bustillo J, et al. Functional imaging of the hemodynamic sensory gating response in schizophrenia. Hum Brain Mapp. 2013;34(9):2302–12. 10.1002/hbm.22065 PMC4020570 22461278

[36] Qureshi MNI, Oh J, Cho D, Jo HJ, Lee B. Multimodal discrimination of schizophrenia using hybrid weighted feature concatenation of brain functional connectivity and anatomical features with an extreme learning machine. Front Neuroinform. 2017;11:59. 10.3389/fninf.2017.00059 PMC5596100 28943848

[37] Calhoun VD, Sui J, Kiehl K, Turner J, Allen E, Pearlson G. Exploring the psychosis functional connectome: aberrant intrinsic networks in schizophrenia and bipolar disorder. Front Psychiatry. 2012;2:75. 10.3389/fpsyt.2011.00075 PMC3254121 22291663

[38] Hanlon FM, Houck JM, Pyeatt CJ, Lundy SL, Euler MJ, Weisend MP, et al. Bilateral hippocampal dysfunction in schizophrenia. Neuroimage. 2011;58(4):1158–68. 10.1016/j.neuroimage.2011.06.091 PMC4063352 21763438

[39] Whitfield-Gabrieli S, Ford JM. Default mode network activity and connectivity in psychopathology. Annu Rev Clin Psychol. 2012;8:49–76. 10.1146/annurev-clinpsy-032511-143049 22224834

[40] Eickhoff SB, Stephan KE, Mohlberg H, Grefkes C, Fink GR, Amunts K, et al. A new SPM toolbox for combining probabilistic cytoarchitectonic maps and functional imaging data. Neuroimage. 2005;25(4):1325–35. 10.1016/j.neuroimage.2004.12.034 15850749

[41] Rolls ET, Huang CC, Lin CP, Feng J, Joliot M. Automated anatomical labelling atlas 3. Neuroimage. 2020;206:116189. 10.1016/j.neuroimage.2019.116189 31521825

[42] Scheperjans F, Hermann K, Eickhoff SB, Amunts K, Schleicher A, Zilles K. Observer-independent cytoarchitectonic mapping of the human superior parietal cortex. Cereb Cortex. 2008;18(4):846–67. 10.1093/cercor/bhm116 17644831

[43] Scheperjans F, Eickhoff SB, Hömke L, Mohlberg H, Hermann K, Amunts K, et al. Probabilistic maps, morphometry, and variability of cytoarchitectonic areas in the human superior parietal cortex. Cereb cortex. 2008;18(9):2141–57. 10.1093/cercor/bhm241 PMC3140197 18245042

[44] Scheperjans F, Palomero-Gallagher N, Grefkes C, Schleicher A, Zilles K. Transmitter receptors reveal segregation of cortical areas in the human superior parietal cortex: relations to visual and somatosensory regions. Neuroimage. 2005;28(2):362–79. 10.1016/j.neuroimage.2005.06.028 16054841

[45] Whitfield-Gabrieli S, Nieto-Castanon A. Conn: a functional connectivity toolbox for correlated and anticorrelated brain networks. Brain Connect. 2012;2(3):125–41. 10.1089/brain.2012.0073 22642651

[46] Jafri MJ, Pearlson GD, Stevens M, Calhoun VD. A method for functional network connectivity among spatially independent resting-state components in schizophrenia. Neuroimage. 2008;39(4):1666–81. 10.1016/j.neuroimage.2007.11.001 PMC3164840 18082428

[47] Benjamini Y, Hochberg Y. Controlling the false discovery rate: a practical and powerful approach to multiple testing. J R Stat Soc: Ser B (Methodol). 1995;57(1):289–300.

[48] Chumbley J, Worsley K, Flandin G, Friston K. Topological FDR for neuroimaging. Neuroimage. 2010;49(4):3057–64. 10.1016/j.neuroimage.2009.10.090 PMC3221040 19944173

[49] Shepherd GM, Yamawaki N. Untangling the cortico-thalamo-cortical loop: cellular pieces of a knotty circuit puzzle. Nat Rev Neurosci. 2021;22(7):389–406. 10.1038/s41583-021-00459-3 PMC9006917 33958775

[50] Lisman JE, Pi HJ, Zhang Y, Otmakhova NA. A thalamo-hippocampal-ventral tegmental area loop may produce the positive feedback that underlies the psychotic break in schizophrenia. Biol Psychiatry. 2010;68(1):17–24. 10.1016/j.biopsych.2010.04.007 PMC3507433 20553749

[51] Haber SN. The primate basal ganglia: parallel and integrative networks. J Chem Neuroanat. 2003;26(4):317–30. 10.1016/j.jchemneu.2003.10.003 14729134

[52] Crapse TB, Sommer MA. Corollary discharge circuits in the primate brain. Curr Opin Neurobiol. 2008;18(6):552–7. 10.1016/j.conb.2008.09.017 PMC2702467 18848626

[53] Sommer MA, Wurtz RH. Brain circuits for the internal monitoring of movements. Annu Rev Neurosci. 2008;31:317–38. 10.1146/annurev.neuro.31.060407.125627 PMC2813694 18558858

[54] Frith CD, Blakemore S-J, Wolpert DM. Explaining the symptoms of schizophrenia: abnormalities in the awareness of action. Brain Res Rev. 2000;31(2–3):357–63. 10.1016/s0165-0173(99)00052-1 10719163

[55] Frith C. The neural basis of hallucinations and delusions. C R Biol. 2005;328(2):169–75. 10.1016/j.crvi.2004.10.012 15771003

[56] van de Ven V, Rotarska Jagiela A, Oertel-Knöchel V, Linden D. Reduced intrinsic visual cortical connectivity is associated with impaired perceptual closure in Schizophrenia. Neuroimage Clin. 2017;15:45–52. 10.1016/j.nicl.2017.04.012 PMC5407639 28480163

[57] Khurana I, Khurana A, Kowlgi NG. Textbook of medical physiology. 3rd edn. India: Elsevier; 2019.

[58] Liddle PF, Friston KJ, Frith CD, Hirsch SR, Jones T, Frackowiak RS. Patterns of cerebral blood flow in schizophrenia. Br J Psychiatry. 1992;160(2):179–86. 10.1192/bjp.160.2.179 1540757

[59] Csernansky JG, Bardgett ME. Limbic-cortical neuronal damage and the pathophysiology of schizophrenia. Schizophr Bull. 1998;24(2):231–48. 10.1093/oxfordjournals.schbul.a033323 9613623

[60] Bogerts B. The temporolimbic system theory of positive schizophrenic symptoms. Schizophr Bull. 1997;23(3):423–35. 10.1093/schbul/23.3.423 9327507

[61] Tlamsa AP, Brumberg JC. Organization and morphology of thalamocortical neurons of mouse ventral lateral thalamus. Somatosens Mot Res. 2010;27(1):34–43. 10.3109/08990221003646736 PMC2839898 20141408

[62] Bernard JA, Mittal VA. Cerebellar-motor dysfunction in schizophrenia and psychosis-risk: the importance of regional cerebellar analysis approaches. Front Psychiatry. 2014;5:160. 10.3389/fpsyt.2014.00160 PMC4243486 25505424

[63] Bédard MA, Agid Y, Chouinard S, Fahn S, Korczyn AD. Mental and behavioral dysfunction in movement disorders. 1st edn. New York: Springer; 2003.

[64] Graves WW, Grabowski TJ, Mehta S, Gupta P. The left posterior superior temporal gyrus participates specifically in accessing lexical phonology. J Cognit Neurosci. 2008;20(9):1698–710. 10.1162/jocn.2008.20113 PMC2570618 18345989

[65] Tang J, Liao Y, Zhou B, Tan C, Liu W, Wang D, et al. Decrease in temporal gyrus gray matter volume in first-episode, early onset schizophrenia: an MRI study. PLoS One. 2012;7(7):e40247. 10.1371/journal.pone.0040247 PMC3388989 22802957

[66] Sun J, Maller JJ, Guo L, Fitzgerald PB. Superior temporal gyrus volume change in schizophrenia: a review on region of interest volumetric studies. Brain Res Rev. 2009;61(1):14–32. 10.1016/j.brainresrev.2009.03.004 19348859

[67] Behrendt R. Hallucinations: Synchronisation of thalamocortical γ oscillations underconstrained by sensory input. Conscious Cogn. 2003;12(3):413–51. 10.1016/s1053-8100(03)00017-5 12941286

[68] Behrendt R-P, Young C. Hallucinations in schizophrenia, sensory impairment, and brain disease: a unifying model. Behav Brain Sci. 2004;27(6):771–87. 10.1017/s0140525x04000184 16035402

[69] Galderisi S, Mucci A, Volpe U, Boutros N. Evidence-based medicine and electrophysiology in schizophrenia. Clin EEG Neurosci. 2009;40(2):62–77. 10.1177/155005940904000206 19534300

[70] Siekmeier PJ, Stufflebeam SM. Patterns of spontaneous magnetoencephalographic activity in schizophrenic patients. J Clin Neurophysiol. 2010;27(3):179–90. 10.1097/WNP.0b013e3181e0b20a PMC3665947 20461010

[71] Lehmann D, Faber PL, Pascual-Marqui RD, Milz P, Herrmann WM, Koukkou M, et al. Functionally aberrant electrophysiological cortical connectivities in first episode medication-naive schizophrenics from three psychiatry centers. Front Hum Neurosci. 2014;8:635. 10.3389/fnhum.2014.00635 PMC4138932 25191252

[72] Llinás RR, Paré D. Of dreaming and wakefulness. Neuroscience. 1991;44(3):521–35. 10.1016/0306-4522(91)90075-y 1754050

[73] Lisman J. Low-frequency brain oscillations in schizophrenia. JAMA Psychiatry. 2016;73(3):298–9. 10.1001/jamapsychiatry.2015.2320 26792643

[74] Marenco S, Stein JL, Savostyanova AA, Sambataro F, Tan HY, Goldman AL, et al. Investigation of anatomical thalamo-cortical connectivity and FMRI activation in schizophrenia. Neuropsychopharmacology. 2012;37(2):499–507. 10.1038/npp.2011.215 PMC3242311 21956440

[75] Jiang Y, Patton MH, Zakharenko SS. A case for thalamic mechanisms of schizophrenia: Perspective from modeling 22q11. 2 deletion syndrome. Front Neural Circuits. 2021;15:769969. 10.3389/fncir.2021.769969 PMC8693383 34955759

[76] Giraldo-Chica M, Rogers BP, Damon SM, Landman BA, Woodward ND. Prefrontal-thalamic anatomical connectivity and executive cognitive function in schizophrenia. Biol Psychiatry. 2018;83(6):509–17. 10.1016/j.biopsych.2017.09.022 PMC5809301 29113642

[77] Woodward ND, Karbasforoushan H, Heckers S. Thalamocortical dysconnectivity in schizophrenia. Am J Psychiatry. 2012;169(10):1092–9. 10.1176/appi.ajp.2012.12010056 PMC3810300 23032387

[78] Tu P-C, Lee YC, Chen YS, Li CT, Su TP. Schizophrenia and the brain’s control network: aberrant within-and between-network connectivity of the frontoparietal network in schizophrenia. Schizophr Res. 2013;147(2–3):339–47. 10.1016/j.schres.2013.04.011 23706416

[79] Avram M, Brandl F, Bäuml J, Sorg C. Cortico-thalamic hypo-and hyperconnectivity extend consistently to basal ganglia in schizophrenia. Neuropsychopharmacology. 2018;43(11):2239–48. 10.1038/s41386-018-0059-z PMC6135808 29899404

[80] Penner J, Osuch EA, Schaefer B, Théberge J, Neufeld R, Menon RS, et al. Higher order thalamic nuclei resting network connectivity in early schizophrenia and major depressive disorder. Psychiatry Res: Neuroimaging. 2018;272:7–16. 10.1016/j.pscychresns.2017.12.002 29247717

[81] Buckner RL, Andrews-Hanna JR, Schacter DL. The brain’s default network: anatomy, function, and relevance to disease. Ann N Y Acad Sci. 2008;1124:1–38. 10.1196/annals.1440.011 18400922

[82] Vatansever D, Menon DK, Stamatakis EA. Default mode contributions to automated information processing. Proc Natl Acad Sci U S A. 2017;114(48):12821–6. 10.1073/pnas.1710521114 PMC5715758 29078345

[83] Galindo L, Bergé D, Murray GK, Mané A, Bulbena A, Pérez V, et al. Default mode network aberrant connectivity associated with neurological soft signs in schizophrenia patients and unaffected relatives. Front Psychiatry. 2017;8:298. 10.3389/fpsyt.2017.00298 PMC5767074 29375404

[84] Slotnick SD, Schacter DL. The nature of memory related activity in early visual areas. Neuropsychologia. 2006;44(14):2874–86. 10.1016/j.neuropsychologia.2006.06.021 16901520

[85] Vertes RP, Linley SB, Hoover WB. Limbic circuitry of the midline thalamus. Neurosci Biobehav Rev. 2015;54:89–107. 10.1016/j.neubiorev.2015.01.014 PMC4976455 25616182

[86] Andrews J, Wang L, Csernansky JG, Gado MH, Barch DM. Abnormalities of thalamic activation and cognition in Schizophrenia. Am J Psychiatry. 2006;163(3):463–9. 10.1176/appi.ajp.163.3.463 16513868

[87] Welsh RC, Chen AC, Taylor SF. Low-frequency BOLD fluctuations demonstrate altered thalamocortical connectivity in schizophrenia. Schizophr Bull. 2010;36(4):713–22. 10.1093/schbul/sbn145 PMC2894601 18990709

[88] Anticevic A, Haut K, Murray JD, Repovs G, Yang GJ, Diehl C, et al. Association of thalamic dysconnectivity and conversion to psychosis in youth and young adults at elevated clinical risk. JAMA Psychiatry. 2015;72(9):882–91. 10.1001/jamapsychiatry.2015.0566 PMC4892891 26267151

[89] Alexander GE, DeLong MR, Strick PL. Parallel organization of functionally segregated circuits linking basal ganglia and cortex. Annu Rev Neurosci. 1986;9(1):357–81. 10.1146/annurev.ne.09.030186.002041 3085570

[90] Horga G, Abi-Dargham A. An integrative framework for perceptual disturbances in psychosis. Nat Rev Neurosci. 2019;20(12):763–78. 10.1038/s41583-019-0234-1 31712782

[91] Heinks-Maldonado TH, Mathalon DH, Houde JF, Gray M, Faustman WO, Ford JM. Relationship of imprecise corollary discharge in schizophrenia to auditory hallucinations. Arch Gen Psychiatry. 2007;64(3):286–96. 10.1001/archpsyc.64.3.286 17339517

[92] Ford JM, Mathalon DH. Electrophysiological evidence of corollary discharge dysfunction in schizophrenia during talking and thinking. J Psychiatr Res. 2004;38(1):37–46. 10.1016/s0022-3956(03)00095-5 14690769

[93] Ford JM, Roach BJ, Faustman WO, Mathalon DH. Out-of-synch and out-of-sorts: dysfunction of motor-sensory communication in schizophrenia. Biol Psychiatry. 2008;63(8):736–43. 10.1016/j.biopsych.2007.09.013 PMC2330266 17981264

[94] Nawani H, Bose A, Agarwal SM, Shivakumar V, Chhabra H, Subramaniam A, et al. Modulation of corollary discharge dysfunction in schizophrenia by tDCS: preliminary evidence. Brain Stimul: Basic Transl Clin Res Neuromodulation. 2014;7(3):486–8. 10.1016/j.brs.2014.01.003 24507573

[95] Pack CC. Eye movements as a probe of corollary discharge function in schizophrenia. ACS Chem Neurosci. 2014;5(5):326–8. 10.1021/cn5000869 PMC4030793 24786597

[96] Richard A, Churan J, Whitford V, O'Driscoll GA, Titone D, Pack CC. Perisaccadic perception of visual space in people with schizophrenia. J Neurosci. 2014;34(14):4760–5. 10.1523/JNEUROSCI.4744-13.2014 PMC6802721 24695696

[97] Feinberg I. Corollary discharge, hallucinations, and dreaming. Schizophrenia Bull. 2011;37(1):1–3. 10.1093/schbul/sbq115 PMC3004198 20929966

[98] Mellem MS, Jasmin KM, Peng C, Martin A. Sentence processing in anterior superior temporal cortex shows a social-emotional bias. Neuropsychologia. 2016;89:217–24. 10.1016/j.neuropsychologia.2016.06.019 PMC5384858 27329686

[99] Rajarethinam, RP, DeQuardo JR, Nalepa R, Tandon R. Superior temporal gyrus in schizophrenia: a volumetric magnetic resonance imaging study. Schizophr Res. 2000;41(2):303–12. 10.1016/s0920-9964(99)00083-3 10708339

